# RETRACTED: Wang et al. Integrated Analysis of Physiological and Transcriptional Mechanisms in Response to Drought Stress in *Scaevola taccada* Seedlings. *Plants* 2026, *15*, 970

**DOI:** 10.3390/plants15131966

**Published:** 2026-06-26

**Authors:** Yaqin Wang, Wenlan Liu, Cunwu Zuo, Yongzhong Luo, Mengting Huang

**Affiliations:** 1 College of Forestry, Gansu Agricultural University, Lanzhou 730070, China; wyq571071@126.com (Y.W.); luoyzhong@gsau.edu.cn (Y.L.); h13349289515@163.com (M.H.); 2 College of Horticulture, Gansu Agricultural University, Lanzhou 730070, China; zuocw@gsau.edu.cn

The journal retracts the article titled “*Integrated Analysis of Physiological and Transcriptional Mechanisms in Response to Drought Stress in Scaevola taccada Seedlings*” [1], cited above.

Following publication, concerns were brought to the attention of the publisher regarding an overlap between this article [1] and an unpublished thesis produced by a different author.

In accordance with the journal’s standard procedures, an investigation was conducted by the Editorial Office and Editorial Board that confirmed a significant overlap of text, figures and data with the prior thesis, without appropriate acknowledgement or permission. Given the extent of the overlap, the Editorial Board has decided to retract this article as per MDPI’s retraction policy.

This retraction was approved by the Editor-in-Chief of the journal *Plants*.

The authors have been informed of this retraction and have indicated their agreement with this decision.
